# Inter-session repeatability of electroretinography and visual evoked potentials of the Celeris system

**DOI:** 10.1007/s10633-025-10045-y

**Published:** 2025-08-10

**Authors:** Xiuying Tang, Carla J. Abbott, Penelope J. Allen, Chi D. Luu

**Affiliations:** 1 Centre for Eye Research Australia, Royal Victorian Eye and Ear Hospital, East Melbourne, VIC 3002 Australia; 2 Department of Surgery (Ophthalmology), University of Melbourne, East Melbourne, VIC 3002 Australia

**Keywords:** Test–retest, Rodent ERG, Scotopic electroretinogram (ERG), Photopic electroretinogram (ERG), Photopic negative response (PhNR), Visual Evoked Potential (VEP)

## Abstract

**Purpose:**

The Celeris system (Diagnosys LLC) offers a streamlined alternative to the gold-standard Espion system for high-throughput electroretinography (ERG) and visual evoked potentials (VEP) in preclinical studies. This study evaluated its inter-session repeatability of ERG and VEP measurements in healthy rodent retinae.

**Methods:**

Twenty-five wild type Brown Norway rats underwent ERG and VEP testing across two sessions. Dark-adapted ERG a- and b-waves (flash stimulus of 10 cd s/m^2^), and the photopic negative response (PhNR) (flash stimulus of 3 cd s/m^2^) were recorded. VEPs (flash stimulus of 3 cd s/m^2^) were recorded from implanted screw electrodes in the skull. Pearson’s correlation coefficient (ρ), Bland–Altman with 95% limits of agreement (LOA) and a novel cumulative distribution plot were used to assess inter-session agreement.

**Results:**

Intersession dark-adapted a-wave showed a correlation of ρ = 0.73, (P < 0.0001), LOA: –106.4 to 113.9 µV, and 90% of measurements fell within − 24.8 to 58.0% variation (90% range). Intersession dark-adapted b-wave amplitudes showed ρ = 0.65, (P < 0.001), LOA: − 402.5 to 437.4 µV, and 90% range: − 33.7 to 72.6%. Inter-session PhNR-B (base-to-trough) and PhNR-P (peak-to-trough) amplitudes showed ρ = 0.66 and 0.72 respectively; LOA: − 26.7 to 31.1 µV and − 98.9 to 100.1 µV respectively; 90% ranges: − 58.2 to 59.3%, and − 45.6 to 60.9%, respectively. Inter-session VEP P2 amplitude showed ρ = 0.67, (P < 0.001), LOA: 78.5–72.8 µV, and 90% range: − 52.2 to 67.9%. Inter-session VEP implicit time showed ρ = 0.48, (P < 0.05), LOA: − 39.4 to 38.3 ms, and 90% range: − 39.2% to 16.7%.

**Conclusion:**

The study reported the inter-session repeatability of the ERG and VEP tests using the Celeris system. These data facilitate the study design and sample size calculation for future preclinical studies using the Celeris, and serve as a reference for future comparison with other systems, protocols and species.

**Supplementary Information:**

The online version contains supplementary material available at 10.1007/s10633-025-10045-y.

## Introduction

Electroretinography (ERG) and visual evoked potentials (VEP) are widely used as non-invasive methods to assess the function of the retina and the visual cortex, respectively [1]. Electrophysiology is a key tool for detecting functional abnormalities in retinal, optic nerve, and visual pathway disorders, particularly when functional changes precede structural or clinical changes. In addition, ERG has been extensively employed in preclinical studies to evaluate the safety and efficacy of novel therapies [2–5].

The International Society for Clinical Electrophysiology (ISCEV) provides standardized clinical protocols for electrophysiological testing to reduce variability and yield comparable results between clinics [6]. In the context of monitoring disease progression, it is vital to establish the inter-session test–retest repeatability of the procedures as this parameter partly defines the sensitivity of the test in detecting functional changes over time.

The Celeris system (Diagnosys LLC) has emerged as an alternative approach to ERG and VEP testing, aiming to streamline workflows in preclinical electrophysiology testing. One key advantage is the simplification of electrode placement and delivery of light stimulation in rodents compared to traditional systems that use a Ganzfeld bowl, thus reducing setup time. The system's integrated electrodes and stimulator also minimize manual labour to increase throughput. Furthermore, the simplification and ease of electrode placement enhances the consistency of electrode placement which reduces the variability of the measurements and therefore improves the sensitivity in detecting functional changes in longitudinal studies.

To date, data on the repeatability of the Celeris system remains lacking. Understanding the inter-session repeatability of the Celeris system is critical for assessing its reliability in distinguishing true physiological changes from normal variability associated with the tests. This is particularly important in longitudinal studies, where consistent and repeatable data are critical for accurate interpretation of the results.

The aim of this study was to evaluate the repeatability of the ERG and VEP recordings using a Celeris system by examining the inter-session repeatability of three common tests, namely, the full field ERG (ffERG), the photopic negative response (PhNR) and the VEP in rodents. These tests provide insights into various aspects of retinal and visual function including photoreceptor activity (ffERG a-wave), inner retinal function (ffERG b-wave), retinal ganglion cell responses (PhNR), and the integrity of the visual pathways (VEP). They are widely used in both clinical and preclinical electrophysiology, providing a useful framework for evaluating the repeatability of the Celeris system under our experimental conditions [1, 4, 7–10]. Furthermore, comparing repeatability across different electrophysiology systems and animal models remains challenging due to variations in absolute response amplitudes between species and experimental setups. This highlights the need for standardized metrics that enable direct comparison of test–retest variability independent of absolute amplitude values. We introduce a cumulative distribution analysis of percentage variability as a novel method for comparing repeatability across different systems and experimental conditions.

## Methods

### Subjects and ethics statement

Twenty-five normally sighted Brown Norway rats aged 9–10 weeks and weighing between 135 and 260 g were the subjects of this study. All procedures were approved and monitored by the Bionics Institute Animal Research Ethics Committee (AREC#18/383AC) and conducted in accordance with the National Health and Medical Research Council’s ‘Australian code for the care and use of animals for scientific purposes’ (2013), the Victorian State (Australia) “Prevention of Cruelty to Animal’s Act” (1986 and amendment) and the ARVO Statement for the Use of Animals in Ophthalmic and Vision Research.

### Experimental design

The ffERG, PhNR and VEP were performed to record responses generated by the left eye from each animal. As the right eye was assigned to a separate surgical study, it was excluded from this repeatability study. The recordings were performed at baseline (session 1) and the same recordings were repeated after one week (session 2). The data were analyzed to determine the inter-session repeatability of each test.

### Anesthesia

All procedures were completed under anesthesia and every effort was made to minimize suffering. The rats were fully anesthetized using a cocktail of Xylazil (6.5 mg/kg) and Ketamine (70 mg/kg) administered intramuscularly. One-half of the original dose was used for maintenance of anesthesia as necessary. Mydriatic eyedrops (0.5% tropicamide and 2.5% phenylephrine, both from Bausch & Lomb, Chatswood, NSW, Australia) were used for pupil dilatation. Body temperatures were maintained with a heat mat set at 35 °C. The level of anesthesia was monitored by breath rate, level of whisker movement and reflexes (foot and cornea/blink). Ocular lubricant was applied to corneas to prevent corneal drying as required. Hartmann’s solution (balanced electrolytes) was used to maintain hydration (1 ml/100 g, SC) administered subcutaneously at the end of the procedures. All animals were monitored during recovery until they were awake and moving about.

### ERG & VEP recordings

Dark-adapted ffERG, PhNR and VEP were recorded using a Celeris system (Diagnosys LLC, Littleton, MA, USA). Signals were filtered between 1–300 Hz for ERGs, PhNR and VEPs. All recordings were conducted under anesthesia. Rats were lightly secured to a stage to ensure stable position for recordings. For ffERG and PhNR recordings, light guide electrodes were used to deliver the light stimulus and obtain the responses. A reference gold cup electrode was placed in the mouth and a ground needle electrode was placed in the tail. For dark-adapted ffERG, rats were dark-adapted overnight (at least 12 h). Retinal responses to a flash stimulus of 10 cd s/m^2^ (DA10) were acquired and averaged across at least 3 trials. After DA10 ERG, rats were light adapted with 10 cd/m^2^ green light for 2 min before commencing the PhNR recordings. PhNR was recorded using a blue light flash stimulus (3 cd s/m^2^) under green light background (10 cd/m^2^). These parameters have been shown to produce a maximal PhNR response [11]. The PhNR amplitude was calculated by both from the baseline (PhNR-B, baseline-to-trough method) or from the peak of the b-wave (PhNR-P, peak-to-trough method) [12–15]. While the PhNR-B amplitude is widely adopted, the PhNR-P amplitude has been shown to be less variable in humans [16]. As such, the repeatability of both PhNR-B and PhNR-P amplitudes was analyzed.

**Fig. 1:**
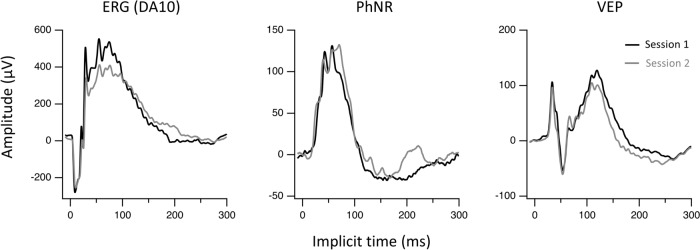
Representative dark-adapted ffERG (DA10), PhNR and VEP response waveforms from two recording sessions in the same animal

For VEP recordings, a screw electrode was implanted on the skull over the area of the right visual cortex (3 mm lateral to Lambda) and secured with dental cement [17]. Implanted screw electrodes were shown to be more reliable than subdermal electrodes [18]. Additionally, You et al. [18] found that early VEP peaks, such as N1 and P2, exhibited significantly lower inter-session variability compared to later peaks. We therefore selected the P2 peak for analysis due to its characteristic waveform [18]. We also measured VEP P2 implicit time, as prior studies indicated that P1, the initial VEP response with a very small amplitude, is more challenging to analyze for implicit time [19]. The screw electrode was left in the skull for follow up VEP recordings. The VEP responses were recorded over the contralateral (right) primary visual cortex as the study measured left eye parameters and over 80% of retinal ganglion cells project to the contralateral cortex [20]. A flash stimulus of 3 cd s/m^2^ was used and the response was averaged over at least 100 trials. Representative ERG, PhNR and VEP response waveforms from 2 recording sessions, one week apart, on the same animal are shown in Fig. 1.

### Data analysis

Statistical analyses were performed using Stata/SE version 18.1 (StataCorp, College Station, Tx) and Microsoft Excel (Microsoft, Corp., Redmond, WA). Data were assessed for normality by Shapiro–Wilk normality tests. For data that passed the normality test, the Pearson’s correlation coefficient (ρ) of DA10 ERG (a-wave, b-wave amplitudes), PhNR amplitude, VEP (amplitude, implicit time) were calculated for each pair of sessions [21]. For data that did not pass the normality test, the Spearman’s correlation coefficient for respective parameters was estimated for each pair of sessions. Scatter and Bland–Altman plots were used to examine the level of inter-session agreement of different ERG and VEP parameters respectively. The limits of agreement (LOA) were determined as ± 1.96 of the mean difference between each pair of sessions multiplied by the standard deviation. The cumulative percentage of difference in measurements between the sessions was plotted to assess the distribution and magnitude of inter-session differences. The percentage inter-session difference in electrophysiology readings was calculated using this equation: (inter-session difference ÷ session1) × 100) [22].

## Results

### Repeatability of ffERG measurements

**Fig. 2:**
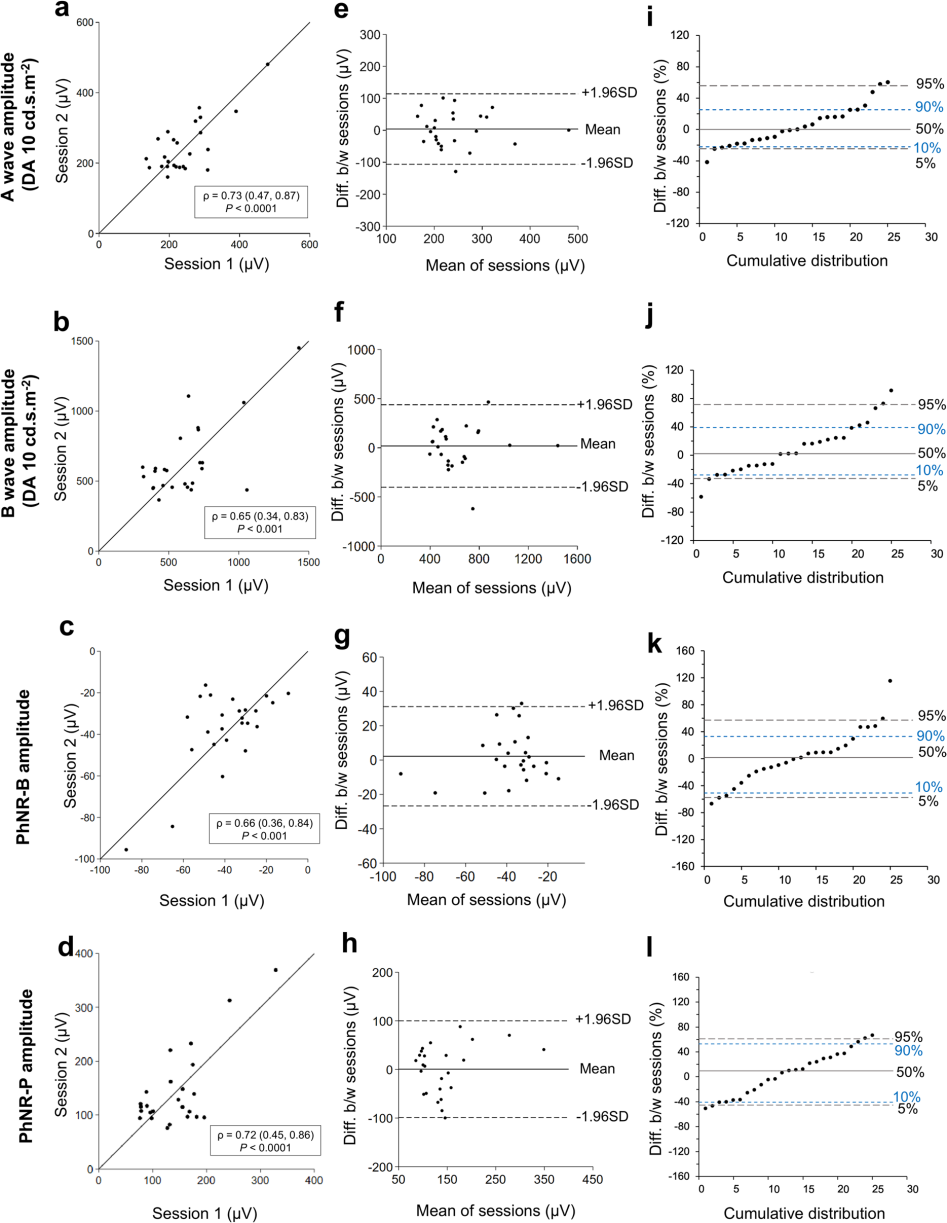
Full-field electroretinography repeatability results. Scatter plots for **a** DA10 ERG a-wave **b** b-wave **c** PhNR-B (**d**) and PhNR-P amplitude. Bland–Altman plots for **e** DA10 ERG a-wave **f** DA10 ERG b-wave **g** PhNR-B and **h** PhNR-P showing the difference between sessions (Diff. b/w sessions) compared to the mean. Cumulative distribution of the percentage difference between sessions for **i** DA10 ERG a-wave, **j** DA10 ERG b-wave **k** PhNR-B and **l** PhNR-P. For the scatter plots, Pearson’s correlation coefficients (ρ) are shown with (95% confidence intervals) and P-values. For Bland–Altman, the mean differences are represented by horizontal grey lines with limits of agreements (± 1.96 SD) represented by horizontal dashed lines. Each circle represents a single measurement. The cumulative distribution shows the 5th, 10th, 50th (solid line), 90th, and 95th percentiles of the inter-session differences. 5th and 95th percentiles are represented by grey horizontal dashed lines, the 10th and 90th percentiles are represented by blue horizontal short, dashed lines

The inter-session agreement for the DA10 ERG a- and b-wave wave responses and PHNR is demonstrated by the scatter, Bland–Altman and cumulative distribution graphs shown in Fig. 2. The DA10 ERG a-wave amplitudes ranged from 134.4 to 480.8 µV. The mean a-wave amplitudes (± standard deviation) were 242.4 ± 76.6 µV for session 1 and 246.1 ± 75.4 µV for session 2. The scatter plot (Fig. 2a) displayed a correlation coefficient of ρ = 0.73 (P < 0.0001). The Bland–Altman plot (Fig. 2e) shows the distribution of the difference in amplitude across sessions compared to the mean, with 95% of the data points having a difference in between sessions of less than 113.9 µV. The limits of agreement were − 106.4 to 113.9 µV. The cumulative distribution plot (Fig. 2i) demonstrated that 90% of all test points were within − 24.8 to 58.0% variation between the two sessions, and 80% of all test points were within − 22.2% to 40.8% variation between the two sessions, suggesting that the intersession variation of the a-wave amplitude is somewhat positively skewed.

DA10 ERG b-wave amplitudes ranged from 313.8 to 1451 µV in total. The mean b-wave amplitudes were 621.3 ± 256.3 µV for session 1, and 638.8 ± 257.8 µV for session 2. The scatter plot (Fig. 2b) showed a correlation coefficient ρ = 0.65 (P < 0.001). The Bland–Altman plot (Fig. 2f) shows that 95% of all data points had a difference between sessions of less than 437.4 µV. The limits of agreement were − 402.5 to 437.4 µV. The cumulative distribution plot (Fig. 2j) demonstrated that 90% of all test points were within a − 33.7 to 72.6% variation between the two sessions and that 80% of all test points were within − 27.7% to 58.1% variation between the two sessions.

PhNR-B amplitudes ranged from − 95.5 to − 9.4 µV across sessions, with a mean of − 39.6 ± 16.7 µV for session 1 and − 37.4 ± 19.0 µV for session 2. The scatter plot (Fig. 2c) showed a correlation coefficient ρ = 0.66 (P < 0.001). The Bland–Altman plot (Fig. 2g) showed that approximately 95% of all data points had a difference between sessions of less than 31.1 µV. The limits of agreement were − 26.7 to 31.1 µV. The cumulative distribution plot (Fig. 2k) demonstrated that 90% of all test points were within − 58.2 to 59.3% variation between the two sessions and that 80% of all test points were within − 51.2 to 47.6% variation between the two sessions.

PhNR-P amplitudes ranged from 76.1 to 369.3 µV across sessions, with a mean of 142.8 ± 58.7 µV for session 1 and 143.3 ± 72.1 µV for session 2. The scatter plot (Fig. 2d) showed a correlation coefficient ρ = 0.72 (P < 0.0001). The Bland–Altman plot (Fig. 2h) showed that approximately 95% of all data points had a difference between sessions of less than 100.1 µV. The limits of agreement were –98.9 to 100.1 µV. The cumulative distribution plot (Fig. 2l) demonstrated that 90% of all test points were within − 45.6 to 60.9% variation between the two sessions and that 80% of all test points were within − 40.7 to 52.9% variation between the two sessions.

### Repeatability of VEP measurements

**Fig. 3:**
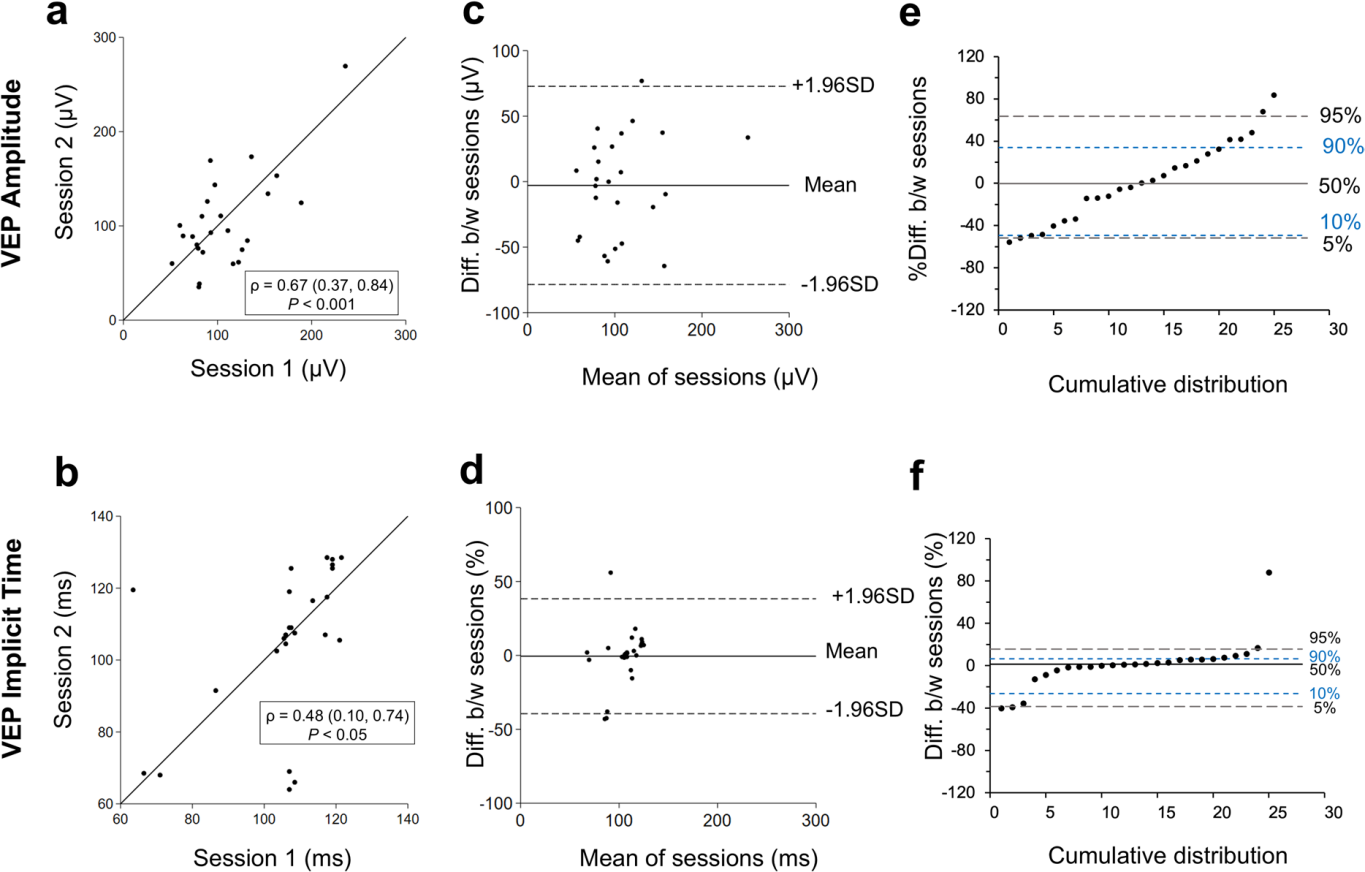
Visual evoked potential repeatability results. Scatter plot for **a** visual evoked potential (VEP) (P2, 3 cd.s.m^−2^) amplitude and **b** implicit time. Bland–Altman plots for **c** VEP amplitude **d** VEP implicit time. Cumulative distribution plots for **e** VEP amplitude and **f** implicit time. For the scatter plots, Pearson’s correlation coefficients (ρ) are shown with (95% confidence intervals) and P-values. For the Bland–Altman and cumulative distribution plots, the mean of session differences is represented by horizontal grey lines with limits of agreements (± 1.96 SD) in horizontal dashed lines. Each circle represents a single measurement. The cumulative distribution shows the 5th, 10th, 50th (solid line), 90th, and 95th percentiles of the intersession differences. 5th and 95th percentiles are represented by grey horizontal dashed lines, the 10th and 90th percentiles are represented by blue horizontal short, dashed lines

The inter-session agreements for VEP P2 response amplitude and implicit time are shown in Fig. 3. VEP P2 response amplitude ranged from 35.18 to 269.5 µV across sessions, with mean (± standard deviation) measurements of 107.8 ± 42.7 µV for session 1 and 104.9 ± 50.5 µV for session 2. The scatter plot (Fig. 3a) showed correlation coefficient ρ = 0.67 (P < 0.001). The Bland–Altman plot (Fig. 3c) showed that approximately 95% of all data points had a difference in between sessions of less than 78.5 µV. The limits of agreement were − 78.5 to 72.8 µV. The cumulative distribution plot (Fig. 3e) demonstrated that 90% of all test points were within − 52.2 to 67.9% variation between the two sessions and that that 80% of all test points were within − 49.3 to 45.1% variation between the two sessions.

VEP P2 implicit time (P2 peak time) ranged from 63.5 to 128.5 ms across sessions. The mean implicit time was 105.3 ± 16.3 ms for session 1 and 104.8 ± 21.5 ms for session 2. The scatter plot (Fig. 3b) demonstrated a correlation coefficient of ρ = 0.48 (P < 0.05). The Bland–Altman plot (Fig. 3d) showed that approximately 95% of all data points had a difference between sessions of < 39.4 ms. The limits of the agreement were − 39.4 to 38.3 ms. The cumulative distribution plot (Fig. 3f) demonstrated that 90% of all test points were within − 39.2 to 16.7% variation between the two sessions and that 80% of all test points were within − 26.4 to 10.5% variation between the two sessions.

## Discussion

This study evaluated the inter-session repeatability of the Celeris system for preclinical research. We provided much needed and important data on the inter-session reproducibility for ffERG, PhNR and VEP tests in a rodent model. The inter-session repeatability data obtained from this study will serve as a reference for future longitudinal studies using the Celeris and provide confidence for its use in evaluating retinal function, efficacy of therapeutic interventions, and disease progression in preclinical models.

While ERG and VEP are well-established techniques, inter-session repeatability data for preclinical models remain sparse, with variability compounded by differences in protocols, animal models, and reporting methods (e.g. coefficients of variability and coefficients of repeatability). Supplementary Table 1 indicates four previous preclinical studies of ERG and/or VEP inter-session repeatability, along with calculations of repeatability for the current study using the same methods as reported in these previous studies, so that context can be given. It is important to acknowledge that various factors contribute to the variability of the measurements. These include the differences in animal models, level of anesthesia, electrode placement, dark adaptation duration and methods, and operators’ discretions. We note that the repeatability data does not necessarily reflect the ERG or VEP system in isolation but the testing procedure in its entirety.

Previous studies used parameters such as coefficient of variation or coefficient of repeatability to determine the inter-session repeatability. These parameters are derived from the absolute response amplitude and therefore, tests or devices that generate a greater amplitude tend to appear more variable because they have a larger amplitude range. Hence, these parameters are not ideal for a direct comparison between tests or devices. In this study, we have used a range of statistical methods to analyze the data. One key novel addition we have in our study is the cumulative distribution plot. Unlike the coefficient of variation and coefficient of repeatability, the cumulative distribution metric is not influenced by the amplitude range generated by each device or test and has a standardized scale (i.e. percentage of difference). Thus, it is a more appropriate parameter for direct comparison of repeatability between devices. Another advantage of using the cumulative distribution plot to compare the variability between tests or devices is the visualization of statistical certainty information from the plot which could help with decision making or setting critical thresholds. For instance, if the cumulative distribution plot shows that 90% of the subjects have an inter-session difference of ± 30%, any longitudinal changes of greater than 30% are likely to be real physiological changes rather than a variation between tests with at least 90% certainty.

Using the data from the cumulative distribution plots, we found that 90% of the subjects had an average inter-session difference in amplitude of approximately 41% for ERG a-wave, 58% for ERG b-wave, 58% for PhNR-B, 55% for PhNR-P, and 60% for VEP. These data suggest that a longitudinal change of more than 50% is required in most of these tests in order to be certain that the changes are physiological. Despite using screw electrodes for VEP and an integrated electrode for ERG recordings to enhance the consistency of electrode placement which reduces the variability, the inter-session variation remains relatively large in this study. This highlights that many factors could contribute to test variability. The large variability observed in our study could arise from methodological and biological factors. For example, in terms of protocol design, subtle differences in anesthesia depth could alter photoreceptor or cortical responses [23]. Biological factors, on the other hand include that retinal ganglion cells exhibit sensitivity to transient changes in intraocular pressure [24]. In light of this, we propose several strategies to improve repeatability. Standardized protocols should be applied to minimize variation in test procedure such as electrode placement, dark or light adaptation and ambient light. Extended signal averaging (increasing trial repetitions) should be considered for low-amplitude parameters. Anesthesia monitoring can also be more rigorous by continuously tracking breath rate and reflexes to minimize physiological fluctuation [25, 26]. Ensuring staff have adequate training and are competent with the test will also improve test repeatability.

Previously, Mortlock et al. found that the peak-to-trough PhNR amplitude is less variable than the baseline-to-trough PhNR amplitude in humans [16]. Interestingly, we found that the variability of PhNR-B (58%) is similar to that of the PhNR-P (55%). The discrepancy between our findings and that of Mortlock is likely due to the absence of baseline drift in rats but present in humans. Unlike humans, where baseline drift significantly impacts PhNR-B repeatability, baseline drift is less impactful in rodents, as controlled experimental settings in rodent studies such as anesthesia reduces spontaneous physiological fluctuations (such as microsaccades and eye movements).

In attempt to compare the repeatability of our ERG and VEP data to that of previous studies, acknowledging that using the coefficient of variation is not ideal, we provided a summary of the data in the Supplementary Table. A longitudinal ERG and VEP study in pigmented rats by Charng et al. [27] reported inter-session coefficient of variations for a- and b-wave that are comparable to the present Celeris study (Supplementary Table 1). Charng et al. used a twin-flash paradigm with a Ganzfeld integrating sphere delivered by stimulation electrodes that are micro-sutured to the sclera. Implanted telemetry transmitters were then used to record ERG signals wirelessly in conscious rats and perform dual-stage amplification. For VEP measurements, the stimulating electrode was implanted 7 mm caudal to bregma. In our study, we used corneal electrodes with integrated stimulators, so that ERG was conducted with stimulation and recording electrodes in one apparatus, and VEP recording electrodes surgically implanted over the visual cortices. Overall, the variability of measurements acquired by the Celeris (13–18% coefficient of variation) is comparable to that acquired by the customised Ganzfield system (15–30% coefficient of variation). Although both studies used a pigmented strain of rat which are likely to have similar visual function, there is some evidence suggesting that the biological differences between species can impact on the reproducibility of electrophysiology recordings [28].

A study by Szabo-Salfay et al. [29] conducted in a single, conscious, albino rat reported a significant discrepancies between sessions for the a-wave amplitude and the VEP amplitude, but not the b-wave amplitude or the VEP latency. This contrasts with our study, where we observed no statistically significant differences in ERG a-wave and VEP amplitude between session 1 and session 2. In Szabo-Salfay et al. study, dark-adapted ERG was conducted in a freely moving rat, using stainless steel screws implanted in the cerebellar, parietal and visual cortices. Signals were recorded using an EEG amplifier (Grass system) and digitised with a CED converter. For VEP, stimulation conditions were the same as that of ERG and recording electrodes were stainless steel cortical screws implanted over the visual cortices. Szabbo-Salfay et al. proposed that the use of freely moving rats was the main driver of the inter-session differences in their study. Conversely, there is some evidence suggesting the use of anesthesia can improve the stability of recordings [30]. Since the Szabo-Salfay et al. repeatability study was conducted in one rat, we were unable to compute a comparable CV% and COR for their study since the variability caused by larger sample size could not be accounted for. In contrast, our CV% accounts for the inter-animal variability within a sample size of 25 animals.

A study by You et al. on the reproducibility of VEP measurements in albino Sprague–Dawley rats [19] using both a PS33-PLUS strobe stimulator (Grass System) and a custom mini-Ganzfeld stimulator system with light emitting diodes report similar coefficients of variation to our Celeris study for P2 VEP amplitude and implicit time with both stimulator systems (Supplementary Table 1; all 20–40% for VEP amplitude, all 4–8% for VEP implicit time). However, the difference in the coefficient of repeatability for VEP amplitude is notable, with their value being 27.7% and 33.2% for their respective stimulators and ours being double at 61.5%. You et al. used implanted screw electrodes at the visual cortex for recordings, similar to the implanted screw electrodes in our Celeris study. The strobe photostimulator used by You et al. had a flash strength of 3 cd s/m^2^, which is the same as for our Celeris study [31]. Our higher COR for VEP amplitude may reflect greater variability introduced by our method of light delivery with the light-guided electrodes compared to You et al.’s custom made LEDs within the Mini-Ganzfeld. A higher COR could be also attributed to significantly higher mean VEP amplitudes (106.4 μV) compared to You et al. (41.65 μV). This results in our CV% being lower given that the calculation for CV% is relative to the mean, whilst our COR may appear higher due to the COR not accounting for the value of the mean. On the other hand, the sample size of the You et al. study (n = 21) was somewhat smaller than ours (n = 25), which lowers their COR (due to lower standard deviation), albeit this lowering effect is to a lesser extent than the elevation effect of the mean on the COR, leading to their lower COR but higher CV%.

The inter-session repeatability of ERG measures with the Espion E3 system (Diagnosys LLC) and jet electrodes (Universo S.A.) has been performed in anesthetised rabbits by Yip et al. [32]. The coefficient of variation for both a- and b-wave amplitudes were similar to our Celeris study in rats (Supplementary Table 1; all 5–21%). However, the coefficient of repeatability was greater in our study, which may indicate greater within-animal differences in our current study and reflect a greater stability of response in larger eyes given that rabbit eyes are about 3 times larger than rat eyes. However, the mean amplitude of our a-wave (244 μV) and b-wave (630 μV) are higher than that for Yip et al. (100 μV for a-wave and 320 μV for b-wave). This may result in their lower COR as aforementioned. The use of cumulative distribution plot to examine the levels of repeatability in our study overcomes the limitations associated with the use of the absolute amplitude parameter as discussed above.

The limitations of our study include a small sample size of 25 rats; however, this sample size is common for preclinical studies. The study was limited to a single rodent species (Brown Norway rats). While this strain is widely used in preclinical studies, the generalizability of our findings to other species (e.g., mice and albino rats) remains to be established. Celeris system’s integrated electrodes are currently tailored to rodent ocular anatomy (ocular size and curvature), limiting immediate application to non-rodent models without technical modifications. Future validation studies could assess the Celeris system’s repeatability in mice and albino rats (e.g., using the novel cumulative distribution analysis). While inter-ocular variability could be valuable in cross-sectional designs (e.g., using one eye as a control), our focus on longitudinal reliability prioritizes consistency within the same eye across sessions, which is critical for longitudinal studies where the same eye is tracked over time (e.g., treatment efficacy or disease progression). Conversely, for experiments requiring paired-eye comparisons (e.g., unilateral treatment with the contralateral eye as control) [33, 34], we acknowledge that inter-eye variability should be characterized. However, such variability is distinct from inter-session repeatability and may depend on factors like systemic effects or bilateral disease models [35]. Future studies could address this by including inter-eye correlation metrics if both eyes are available. Intra-session repeatability (i.e., immediate test–retest variability within the same session) was not evaluated due to ethical and practical constraints. Repeated recordings within a single session would require prolonged anesthesia and re-dark adaptation, and confound results given pre-clinical workflows. Nevertheless, the intra-session variability could be minimized with adequate number of trials for averaging. Finally, although it would be beneficial to compare the Celeris system with other established platforms, this is beyond the scope of this study because we do not have access to other systems. However, we have provided a unique standardized repeatability metric in the form of cumulative distribution which could be used for a direct comparison between the Celeris with other systems when the data from the other systems become available in the future.

In conclusion, this study establishes the inter-session repeatability of ERG and VEP in rats using the Celeris system. While general comparisons with prior studies are provided, direct cross-system evaluations are limited by methodological differences between studies. Our cumulative distribution data provide baseline variability thresholds for the Celeris system, enabling future studies to benchmark protocols and calculate sample sizes based on parameter-specific variability. These findings will refine testing workflows and guide robust longitudinal study design.

## Supplementary Information

Below is the link to the electronic supplementary material.

Supplementary file1 (DOCX 27 KB)

## Data Availability

The datasets generated during current study are available from the corresponding author on reasonable request.
