# Supplementary material for: Inter-session repeatability of electroretinography and visual evoked potentials of the Celeris system
Source: Doc Ophthalmol. 2025 Aug 10;151(2):133–43. doi: 10.1007/s10633-025-10045-y (PMC12436515; doi:10.1007/s10633-025-10045-y)
Supplement: Supplementary file 1 — Supplementary file1 (DOCX 27 KB) [file 10633_2025_10045_MOESM1_ESM.docx]

***Supplementary Information***

***Table 1 Comparison of inter-session repeatability across different preclinical ERG systems*** Measures of repeatability calculated in accordance with methods used by different preclinical studies to make comparisons of differences in repeatability between other systems with the Celeris system used in the present study. All values of coefficient of variation have been converted to percentage. Abbreviation: σw, within-subject standard deviation. Mean refers to overall mean.

| **Study** | **Animal** | **ERG system & electrode used** | **Measure of Repeatability** | **Calculation Method** | **Parameter** | **Their result** | **Our result** |
| --- | --- | --- | --- | --- | --- | --- | --- |
| Charng et al [1] | Rat (Long-Evans, pigmented), conscious | Ganzfeld integrating sphere, wireless transmission from transmitter to receiver. Subconjunctival implanted or AgCl electrodes | *Coefficient of variation (CV%) | $\frac{\sigma w x 100}{mean}$ | a-wave amplitude | 15%  (for AgCl)  30%  (for implanted) | 13.1% |
|  |  |  |  |  | b-wave amplitude | 15%  (for AgCl)  40%  (for implanted) | 18.3% |
| You et al [2] | Rat (Sprag-Dawley, albino), anesthetized | Strobe stimulator or Mini-Ganzfeld stimulator (custom made LEDs), with skull-implanted stainless-steel screws | *Coefficient of variation (CV%) | $\frac{\sigma w x 100}{mean}$ | VEP amplitude | 30%  (for Strobe stimulator)  40%  (for Mini-Ganzfeld) | 20.9% |
|  |  |  | Coefficient of Repeatability (COR) | $2.77 x \sigma w$ |  | 27.7  (for Strobe stimulator)  33.2  (for Mini-Ganzfeld) | 61.5 |
|  |  |  | *Coefficient of variation (CV%) | $\frac{\sigma w x 100}{mean}$ | VEP implicit time | 4%  (for Strobe stimulator)  7%  (for Mini-Ganzfeld) | 8.0% |
|  |  |  | Coefficient of Repeatability (COR) | $2.77 x \sigma w$ |  | 19.3  (for Strobe stimulator)  36  (for Mini-Ganzfeld) | 23.5 |
| Yip et al [3] | Rabbit (New Zealand, albino), anesthetized | Espion E3 system with Custom-built electrode (cable of ERG-jet electrode) | Coefficient of variation (CV%) | $\frac{\sigma w x 100}{mean}$ | a-wave amplitude | 21.4% | 13.1% |
|  |  |  | Coefficient of Repeatability (COR) | $2.77 x \sigma w$ |  | 13.5 | 88.8 |
|  |  |  | Coefficient of variation (CV%) | $\frac{\sigma w x 100}{mean}$ | b-wave amplitude | 5.4% | 18.3% |
|  |  |  | Coefficient of Repeatability (COR) | $2.77 x \sigma w$ |  | 21.27 | 319.2 |

*Assumed to have used the standard method of calculation unless specified in the paper.

**References**

[1] J. Charng *et al.*, "Conscious wireless electroretinogram and visual evoked potentials in rats," (in eng), *PLoS One,* vol. 8, no. 9, p. e74172, 2013, doi: 10.1371/journal.pone.0074172.

[2] Y. You, A. Klistorner, J. Thie, and S. L. Graham, "Improving reproducibility of VEP recording in rats: electrodes, stimulus source and peak analysis," *Doc Ophthalmol,* vol. 123, no. 2, pp. 109-119, 2011/10/01 2011, doi: 10.1007/s10633-011-9288-8.

[3] Y. W. Y. Yip, T. C. Man, C. P. Pang, and M. E. Brelén, "Improving the quality of electroretinogram recordings using active electrodes," *Exp Eye Res,* vol. 176, pp. 46-52, 2018/11/01/ 2018, doi: https://doi.org/10.1016/j.exer.2018.06.007.
